# Unraveling the hepatic stellate cells mediated mechanisms in aging's influence on liver fibrosis

**DOI:** 10.1038/s41598-024-63644-1

**Published:** 2024-06-12

**Authors:** Reham M. Wahid, Nancy Husseiny Hassan, Walaa Samy, Amina A. Abdelhadi, Sara F. Saadawy, Sherein F. Elsayed, Sara G. Seada, Sara Refaee Abdo Mohamed

**Affiliations:** 1 Medical Physiology Department, Faculty of Medicine, Zagazig University, Zagazig, Egypt; 2 Human Anatomy and Embryology Department, Faculty of Medicine, Zagazig University, Zagazig, Egypt; 3 Medical Biochemistry Department, Faculty of Medicine, Zagazig University, Zagazig, Egypt; 4 Medical Microbiology and Immunology Department, Faculty of Medicine, Zagazig University, Zagazig, Egypt; 5 Tropical Medicine Department, Faculty of Medicine, Zagazig University, Zagazig, Egypt

**Keywords:** Aging, Liver fibrosis, TGF- β, Desmin, a-SMA, Hepatic stellate cells, Biochemistry, Immunology, Molecular biology, Physiology, Anatomy, Diseases, Gastroenterology

## Abstract

Aging enhances numerous processes that compromise homeostasis and pathophysiological processes. Among these, activated HSCs play a pivotal role in advancing liver fibrosis. This research delved into how aging impacts liver fibrosis mechanisms. The study involved 32 albino rats categorized into four groups: Group I (young controls), Group II (young with liver fibrosis), Group III (old controls), and Group IV (old with liver fibrosis). Various parameters including serum ALT, adiponectin, leptin, and cholesterol levels were evaluated. Histopathological analysis was performed, alongside assessments of TGF-β, FOXP3, and CD133 gene expressions. Markers of fibrosis and apoptosis were the highest in group IV. Adiponectin levels significantly decreased in Group IV compared to all other groups except Group II, while cholesterol levels were significantly higher in liver fibrosis groups than their respective control groups. Group III displayed high hepatic expression of desmin, α-SMA, GFAP and TGF- β and in contrast to Group I. Increased TGF-β and FOXP3 gene expressions were observed in Group IV relative to Group II, while CD133 gene expression decreased in Group IV compared to Group II. In conclusion, aging modulates immune responses, impairs regenerative capacities via HSC activation, and influences adipokine and cholesterol levels, elevating the susceptibility to liver fibrosis.

## Introduction

Numerous biochemical and cellular processes in humans and other organisms are compromised during aging, contributing to time-dependent and progressive pathophysiological and homeostatic dysfunctions^1^. It increases the risk of hepatic damage since it predisposes to liver structure and function deterioration, in addition to inducing a variety of alterations in hepatic cells^2^. There is a 20 to 40% decrease in liver volume in the elderly population, with the extent varying based on sex. Additionally, hepatocytes in the elderly exhibit reduced volume, elevated levels of lipofuscin and secondary lysosomes, and a higher prevalence of polyploidy^3–5^. Cellular senescence is widely acknowledged as the principal age associated phenotypic alteration^6^. The morphological analysis of human livers indicated the presence of pseudo-capillarization and signs of de-differentiation in liver sinusoidal endothelial cells. Additionally, there was a slight activation of HSCs in human-aged livers^7^. Liver aging is characterized by diminished metabolic and proliferative functions, as well as an elevated risk to develop chronic liver diseases^8^.

Liver fibrosis is a highly progressive liver disease that occurs due to chronic liver inflammation caused by non-alcoholic steatohepatitis, excessive alcohol consumption, autoimmune liver diseases, and infectious agents, and it is characterized by the excessive accumulation of extracellular matrix (ECM) proteins such as collagen^9^. Fibrotic conditions are conceivably reversible after the elimination of their etiological sources. However, significant percentages of patients with advanced cirrhosis did not experience disease regression^10^.

Activated HSCs are crucial for the development of fibrotic tissue during liver fibrosis progression. HSCs comprise 10% of all resident cells in the perisinusoidal space of the liver. HSCs are quiescent (qHSCs) in healthy liver expressing platelet-derived growth factor receptor, lecithin retinol acyltransferase (LRAT), desmin, and lipid droplets containing vitamin A^11^. During healthy aging, HSCs display a subtle but notable activation state. This is demonstrated by increased proliferation and a significantly increased expression of specific markers, such as smooth muscle actin (α-SMA), collagen I, and collagen IV. In addition, there is an elevation in inflammatory, senescence, and pro-oxidant markers, as well as evidence of matrix accumulation within the parenchyma or sinusoids^12^. Interstingly, in chronic liver disease, the aged HSCs exhibit increased intracellular lipid deposition. This is likely due to changes in retinoid metabolism and lipid breakdown, as demonstrated by an increase in the number and size of lipid droplets by electron microscopy^13,14^.

Upon activation by different inflammatory cytokines and mediators, HSCs are phenotypically converted into profibrogenic myofibroblasts (activated)^15^. The activation of HSCs results in the loss of cytoplasmic vitamin A droplets. They also have a stretched shape and contractile characteristics and exhibit variable transcriptional features^16^. Studies on the pathogenesis of hepatic fibrosis have focused on mechanisms causing HSC activation^11^.

Recent reports indicate that a novel subset of rat HSCs expresses CD133, also known as prominin-1 (PROM1), and exhibits progenitor cell characteristics. There is a suggestion that chronically injured livers have an increased expression of CD133 + HSCs^17^. CD133 (PROM1) deficiency demonstrated exaggerated experimental liver fibrosis. This is accompanied by enhanced transforming growth factor-β (TGF-β) signaling, which is caused by reducing SMAD7 protein expression in hepatocytes. Therefore, CD133 (PROM1) plays a crucial role in preventing liver fibrosis through its negative regulation of TGF- β^18^, which is considered the most potent fibrogenic cytokine^19^.

The pathophysiology of liver fibrosis and chronic tissue inflammation occurring in response to liver injury is widely known to be regulated by various types of inflammatory immune cells^20^. Forkhead Box P3 (Foxp3) regulatory T (Treg) cells play a crucial role in maintaining tissue homeostasis and immunological self-tolerance^21^. Treg cells migrate to the liver in order to regulate tissue inflammation and pathology in various pathogenic circumstances in both human and animal models^22–24^. Nevertheless, there is limited data regarding the mechanisms by which Tregs regulate long-term hepatic fibrosis and inflammation^25^. HSCs have been linked to the synthesis of TGF- β and all-trans retinoic acid (ATRA), both necessary for Treg differentiation^26^.

In the present study, we aim to elucidate the effect of aging on liver fibrosis and to investigate the role of HSCs as a potential therapeutic option for liver fibrosis.

## Results

### Effect of induction of liver fibrosis on serum level of cholesterol, leptin, adiponectin, and alanine transferase (ALT)

The mean values of serum cholesterol, ALT, and leptin levels were significantly higher in liver fibrosis-induced Group II and Group IV compared to normal groups ( *P* < *0.001),* as presented in (Table 1).

**Table 1:** Serum assessment of ALT, cholesterol, adiponectin, and leptin in all studied groups.

	Group 1	Group 2	Group 3	Group 4	F	*P* value
ALT	89.65 ± 4.6	127.25 ± 7.46^a^	116.4 ± 6.06^a b^	141.04 ± 7.22^a b c^	87.82	< 0.001 **
Cholesterol	76.86 ± 6.86	110.26 ± 5.11^a^	114.7 ± 27.31^a^	152.17 ± 34.07^a b c^	15.05	< 0.001 **
Adiponectin	2.89 ± 0.34	1.57 ± 0.41^a^	2.3 ± 0.52^a b^	1.18 ± 0.53^a c^	20.83	< 0.001**
Leptin	2.97 ± 0.37	4.84 ± 0.67^a^	1.3 ± 1.05^a b^	3.23 ± 1.99^b c^	12.72	< 0.001 **

All continuous data were expressed using mean (± SD) where:

*P* value ≤ 0.001** was considered statistically highly significant(S).

^a^ means: significant compared with group 1.

^b^ means: significant compared with group 2.

^c^ means : significant compared with group 3.

In contrast, the serum level of adiponectin decreased after liver fibrosis induction. There was a highly significant decrease in the mean value of serum adiponectin level in liver fibrosis-induced groups II and IV compared to normal groups I and III, respectively (*P* < *0.001),* as shown in (Table 1)**.**

### Histopathological assessment results

#### Hematoxylin and eosin staining results

**Figure 1:**
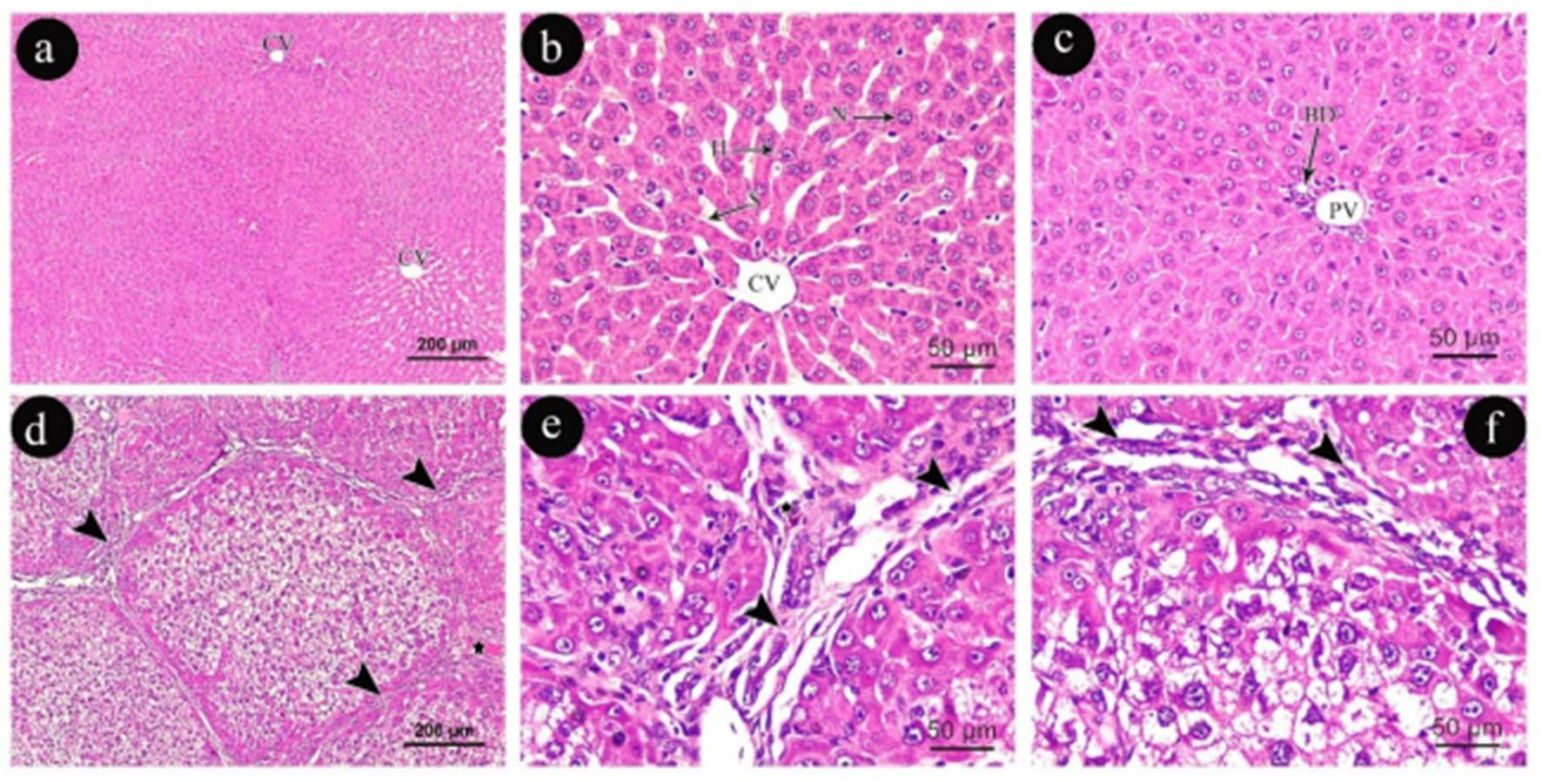
Photomicrographs of liver sections (**a**, **b** & **c**) in group (I) show; CV: central vein, H: polygonal hepatocyte, N: nucleus of hepatocyte, S: blood sinusoids, PV: portal vein & BD: bile duct. Photomicrographs of liver sections (**d**, **e** &**f**) in group (II) show; a well-developed area of fibrosis with cellular infiltration (arrow heads) and areas of congestion (star). (**a**, **d**: H&E × 100, scale 200um, **b**, **c**, **e**, **f**: H&E × 400, scale 50um).

The examined sections of the control rats showed the normal structure of the liver lobes, with cords of polygonal acidophilic hepatocytes that had well-preserved vesicular nuclei. (Fig. 1a&b) shows the presence of narrow blood sinusoids with a thin wall between the hepatocyte cords and the endothelium lining. A bile duct is lined with cuboidal cells, and a portal vein with a thin wall is present in the portal region (Fig. 1c).

In contrast, hepatocytes in Group (II) sections underwent hemorrhagic necrosis and apoptosis. Thick collagen fibers with well-developed fibrosis and early cirrhosis were clearly visible (Fig. 1d, e, f).

**Figure 2:**
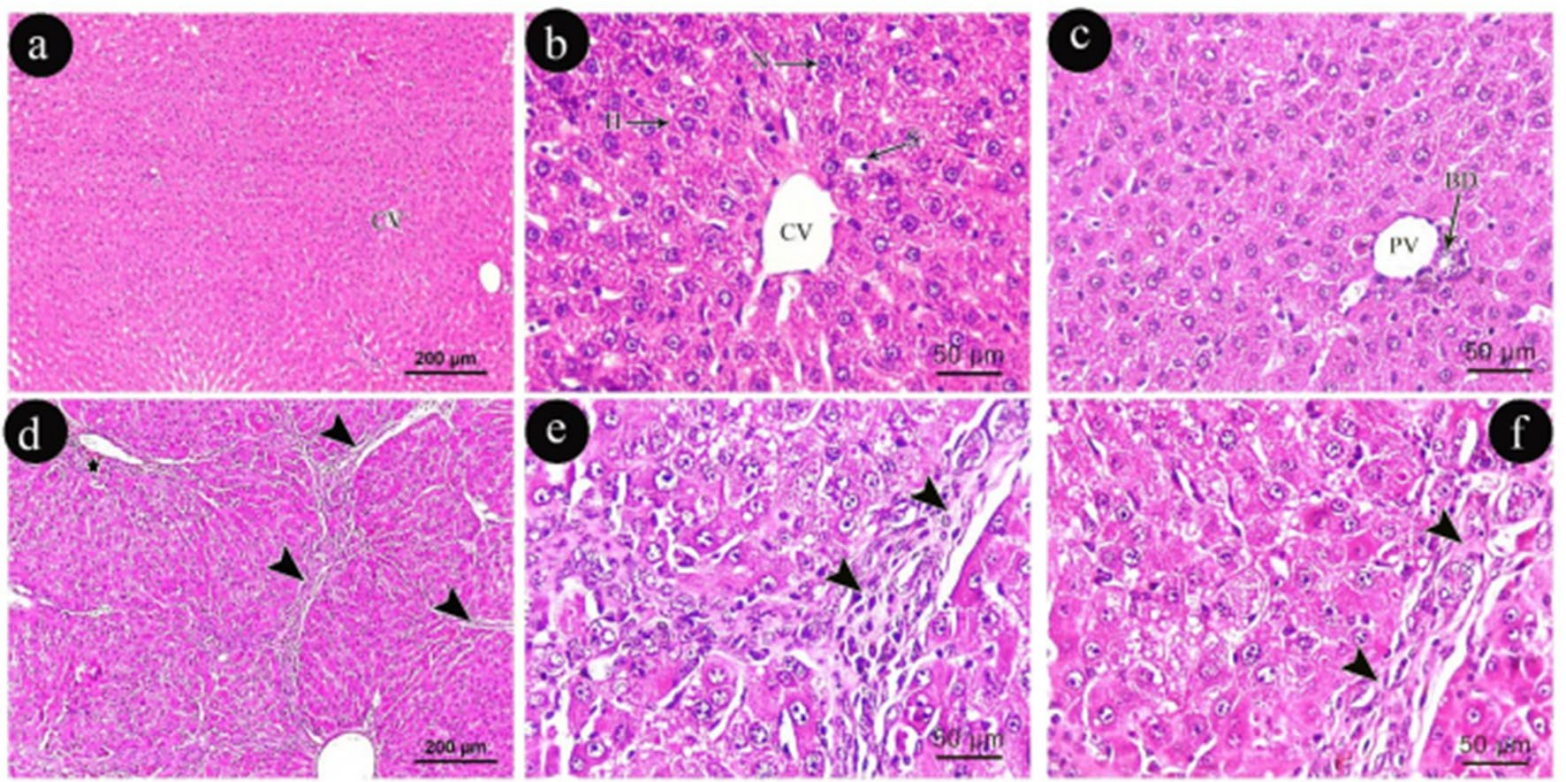
Photomicrographs of liver sections (**a**, **b** & **c**) in group (III) show; CV: central vein, H: polygonal hepatocyte, N: nucleus of hepatocyte, S: blood sinusoids, PV: portal vein & BD: bile duct. Photomicrographs of liver sections (**d**, **e** & **f**) in group (IV) show; a well-developed area of fibrosis with cellular infiltration (arrow heads) and areas of hemorrhage (star). (**a**, **d**: H&E × 100, scale 200um, **b**, **c**, **e**, **f**: H&E × 400, scale 50um).

In addition, Group (III) exhibited a slightly altered hepatic lobular architecture when compared to Group (I) in the form of less pronounced cell boundaries with less distinct contours of sinusoids (Fig. 2 a, b, c). In Group (IV), there was evident cellular damage characterized by the presence of thick collagen fibers, cirrhosis, and fibrosis with cell infiltration and marked intercellular spacing (Fig. 2d, e, f).

#### Masson trichrome (MT) results

**Figure 3:**
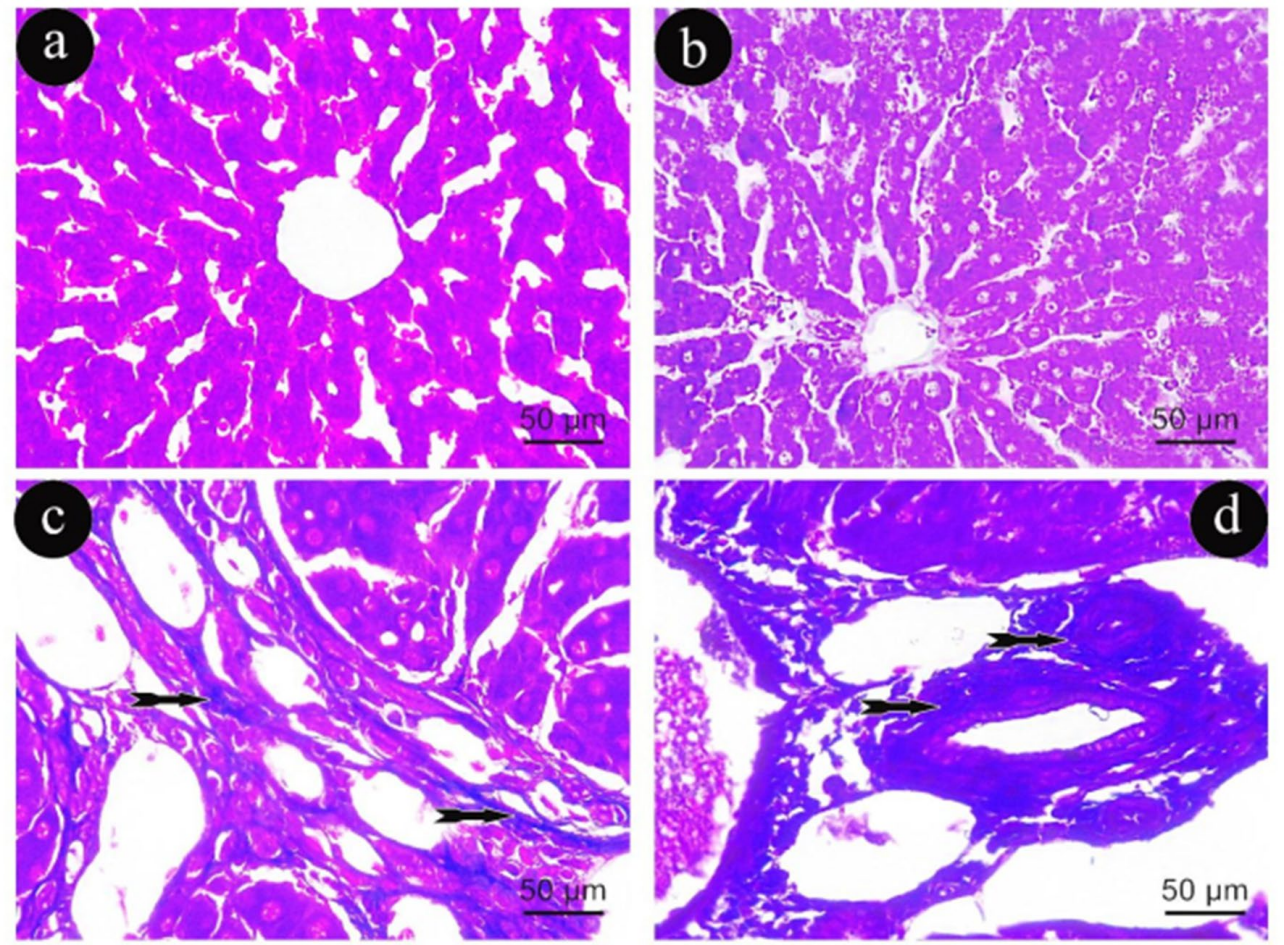
Photomicrographs of liver sections representing the Masson Trichrome staining in group (I) in (**a**&**b**) and (II) in (**c**&**d**). Tailed arrows: blue staining of collagen fibers. (Masson Trichrome × 400, scale 50um).

In Group (I), extremely minimal basophilic collagen fibers surrounding the central and portal veins respectively (Fig. 3a, b). Conversely, Group (II) demonstrated a notable increase in basophilic collagen fibers surrounding the central and portal veins respectively compared with Group (I) (Fig. 3c, d).

**Figure 4:**
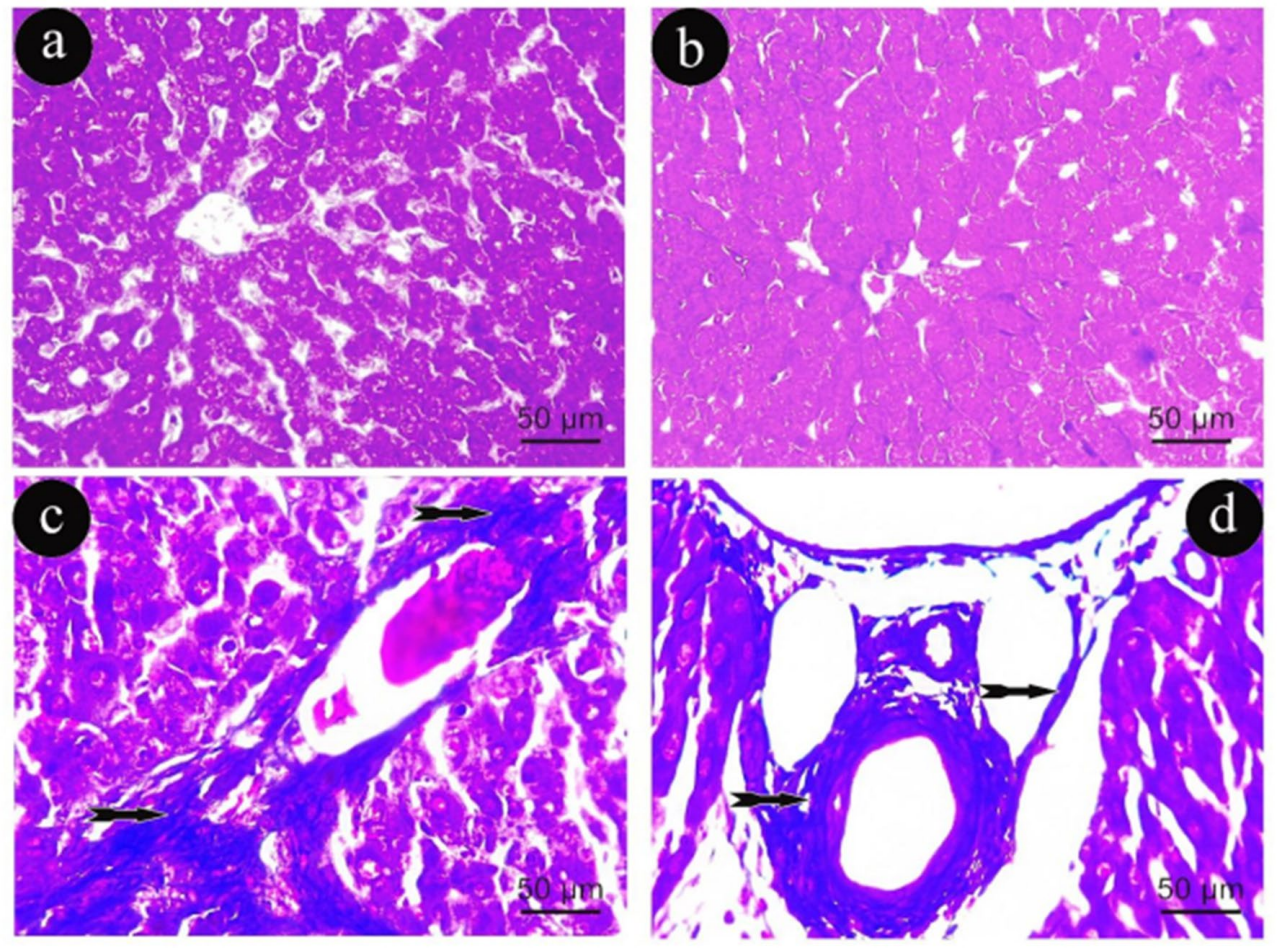
Photomicrographs of liver sections representing the Masson Trichrome staining in group (III) in (**a**&**b**) and (IV) in (**c**&**d**). Tailed arrows: blue staining of collagen fibers. (Masson Trichrome × 400, scale 50um).

In Group (III), there was a slight basophilic collagen fibers surrounding the central and portal veins respectively, as compared to Group (I) (Fig. 4a, b). In contrast, in Group (IV), there was an observed increase in basophilic collagen fibers compared with Group (III) (Fig. 4c, d).

#### Immune-histochemistry results

- Desmin immune-histochemical staining

**Figure 5:**
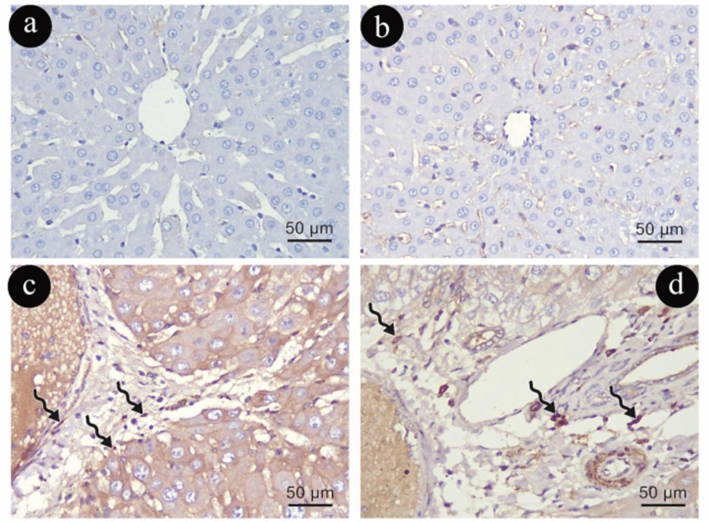
Photomicrographs representing desmin immune -histochemistry staining of liver sections in group (I)in (**a**&**b**) and (II) in (**c**&**d**). Zigzag black arrows: positive cytoplasmic immune reaction of desmin in (HSC). (Desmin × 400, scale 50um).

In Group (I), HSCs had a normal distribution and were rarely observed in Desmin immune-reactive cytoplasmic reaction in the smooth muscle cells of blood vessel walls, in a small number of myofibroblasts of the portal stroma, and in specific cells found in zones 1 and 3 of the liver lobules (Fig. 5a, b). Conversely, Group (II) demonstrated a notable increase in the number of HSC immune-positive nuclei with increased parenchymal reaction compared with Group (I) (Fig. 5c, d).

**Figure 6:**
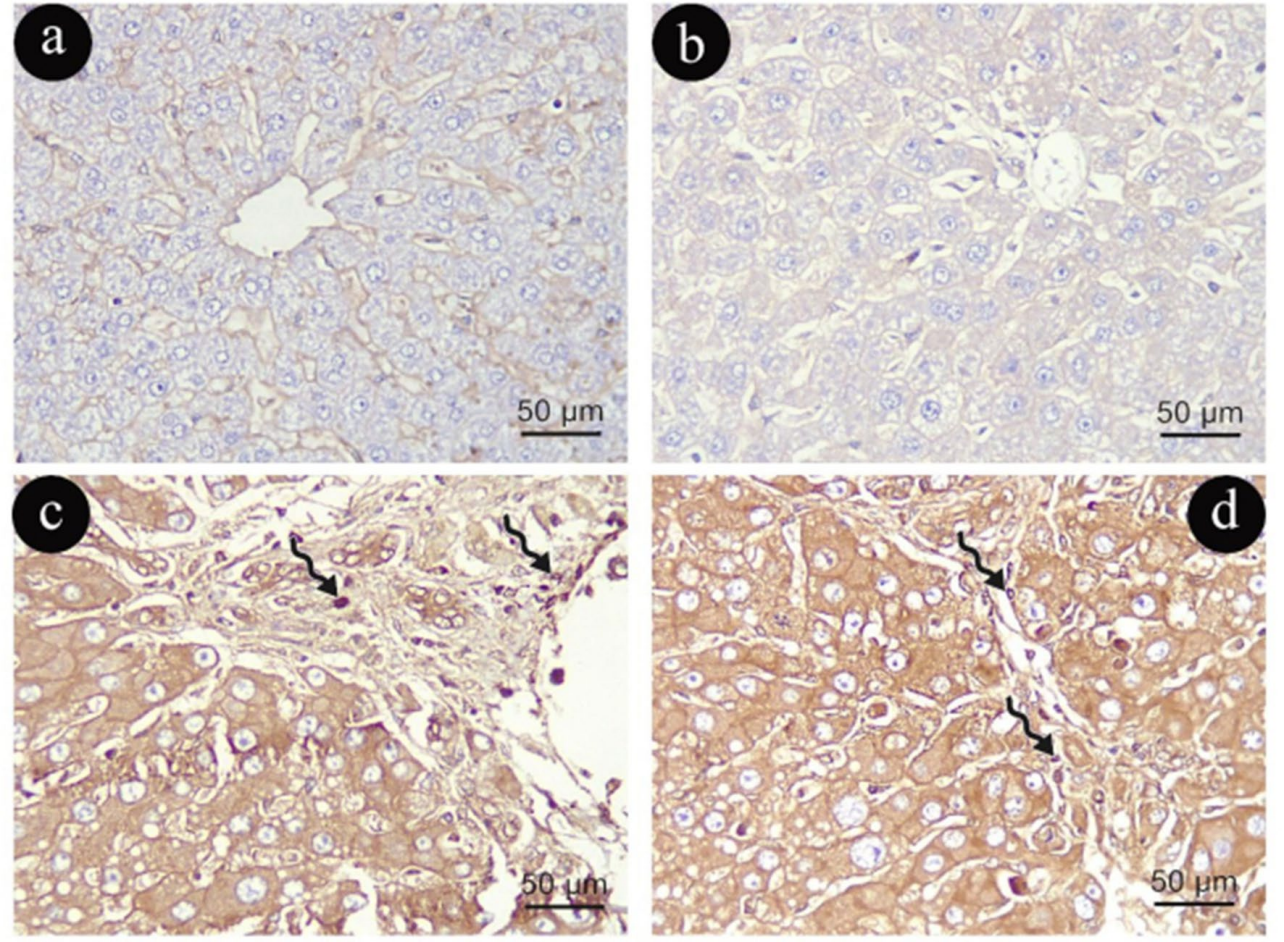
Photomicrographs representing desmin immune -histochemistry staining of liver sections in group (III)in (**a**&**b**) and (IV) in (**c**&**d**). Zigzag black arrows: positive cytoplasmic immune reaction in (HSC). (Desmin × 400, scale 50um).

In Group (III), a slight alteration in the normal distribution of aging HSCs in myofibroblasts located in the interface zone between the portal space and the lobule, where both animal groups' stellate cell populations grew noticeably expressing desmin immune-reactive cytoplasmic reaction, as compared to Group (I) (Fig. 6a, b). In contrast, in Group (IV), there was an observed increase in the number of HSC immune-positive nuclei with increased parenchymal reaction compared with Group (III) (Fig. 6c, d).

- α -SMA immune-histochemical staining

**Figure 7:**
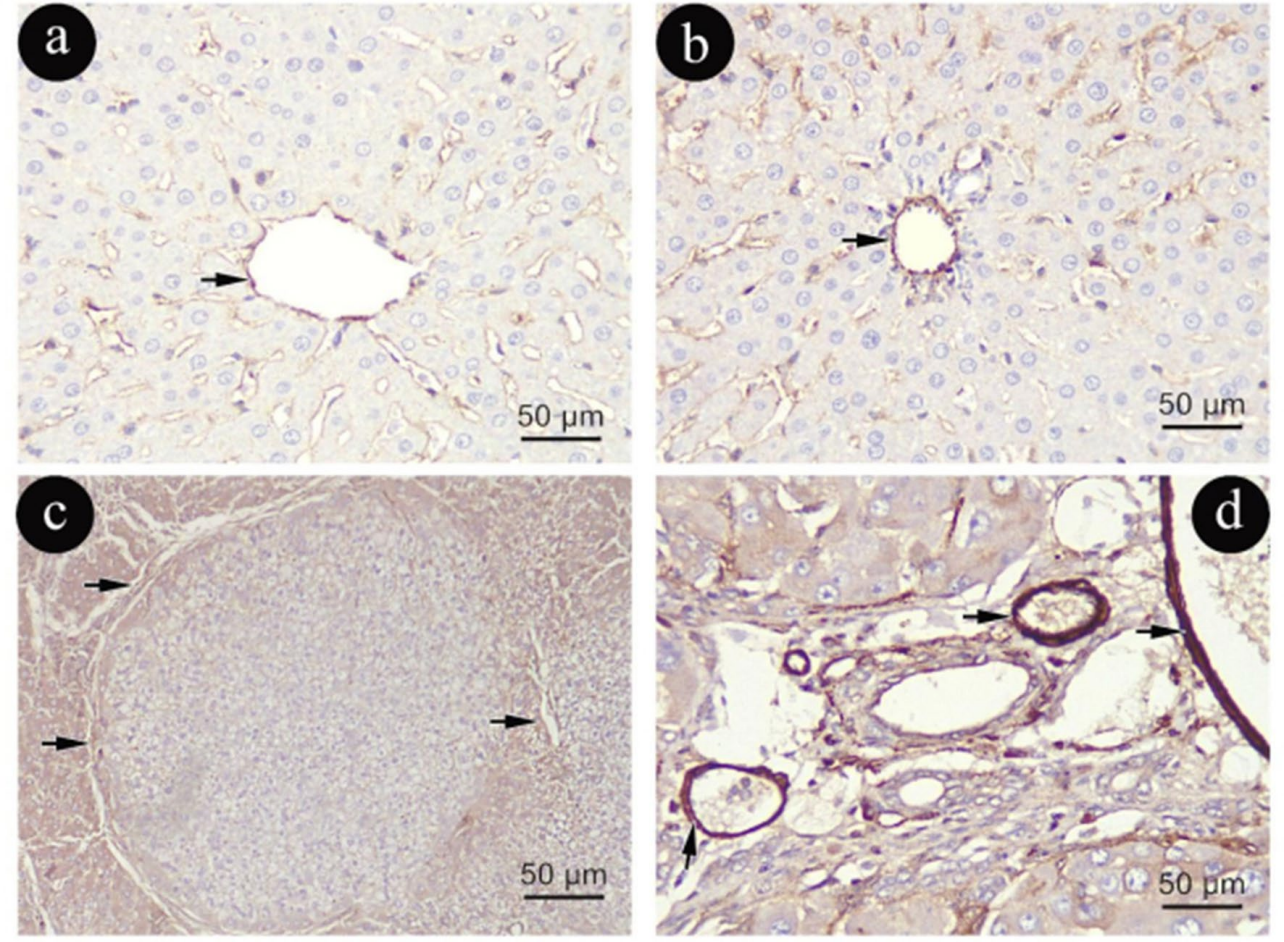
Photomicrographs representing α-SMA immune -histochemistry staining of liver sections in group (I)in (**a**&**b**) and (II) in (**c**&**d**). Short arrows: positive cytoplasmic immune reaction in activated (HSC). (α -SMA X400, scale 50um).

In Group (I), activated HSCs were rarely detectable in α-SMA immune reactivity with normal distribution in the wall of central vein and hepatic artery and portal vein branches (Fig. 7a, b). In contrast to Group (I), Group (II) demonstrated a noticeable increase in activated HSC immune-positive cells in the hepatic artery and portal vein branch walls and blood sinusoids (Fig. 7c, d).

**Figure 8:**
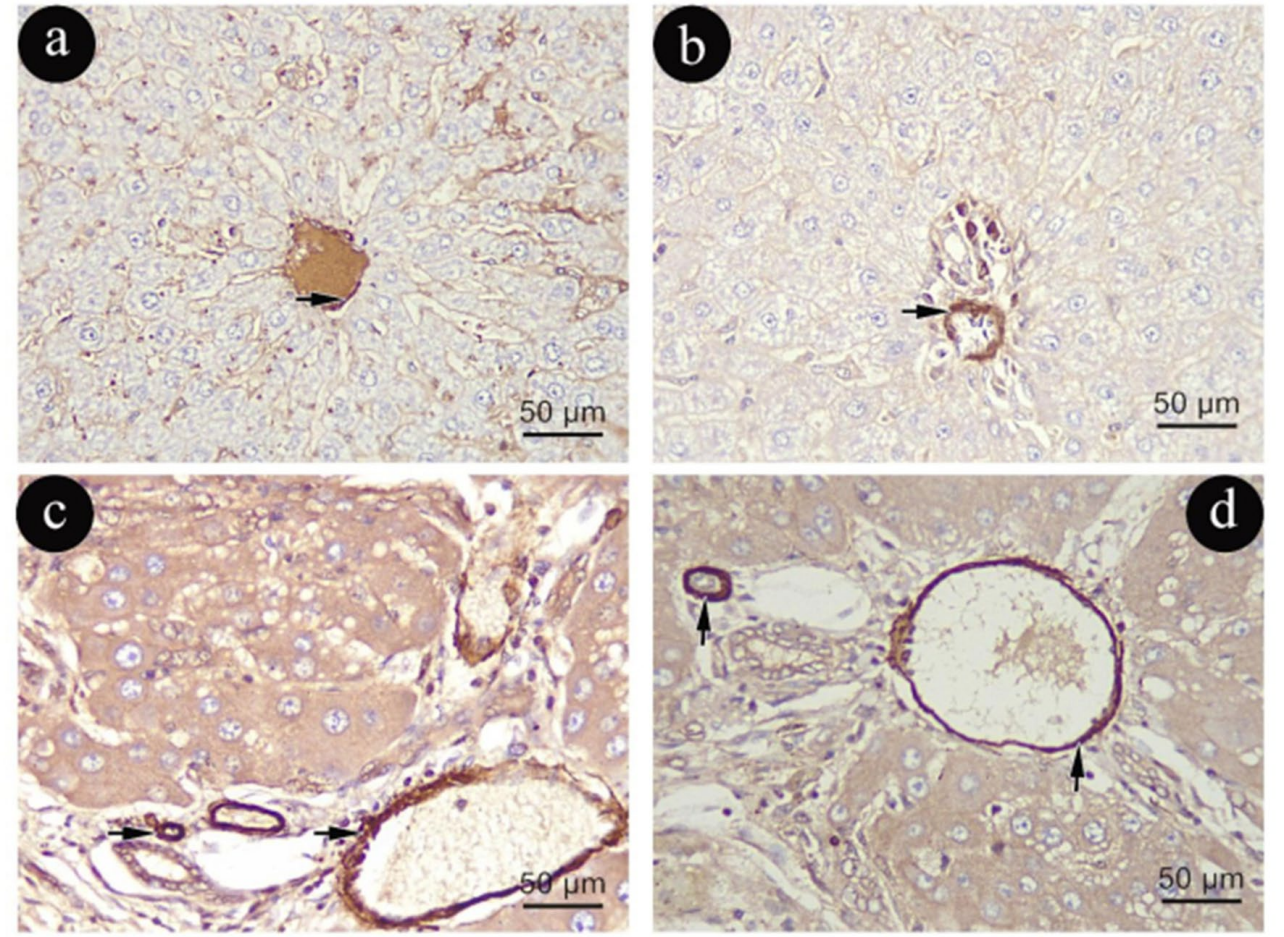
Photomicrographs representing α-SMA immune -histochemistry staining of liver sections in group (III)in (**a**&**b**) and (IV) in (**c**&**d**). Short arrows: positive cytoplasmic immune reaction in activated (HSC). (α -SMA X400, scale 50um).

The distribution of aged HSCs in Group (III) exhibited a slight alteration in the form of myofibroblasts that stained intensely for α-SMA could be easily identified, and they formed layers around the proliferated bile ductules when compared to Group (I), as demonstrated in (Fig. 8a, b). In contrast, Group (IV) exhibited more HSC immune-positive reactions than Group (III) in hepatic arteries and blood sinusoid walls (Fig. 8c, d).

- Glial Fibrillary Acidic Protein (GFAP) immune-histochemical staining

**Figure 9:**
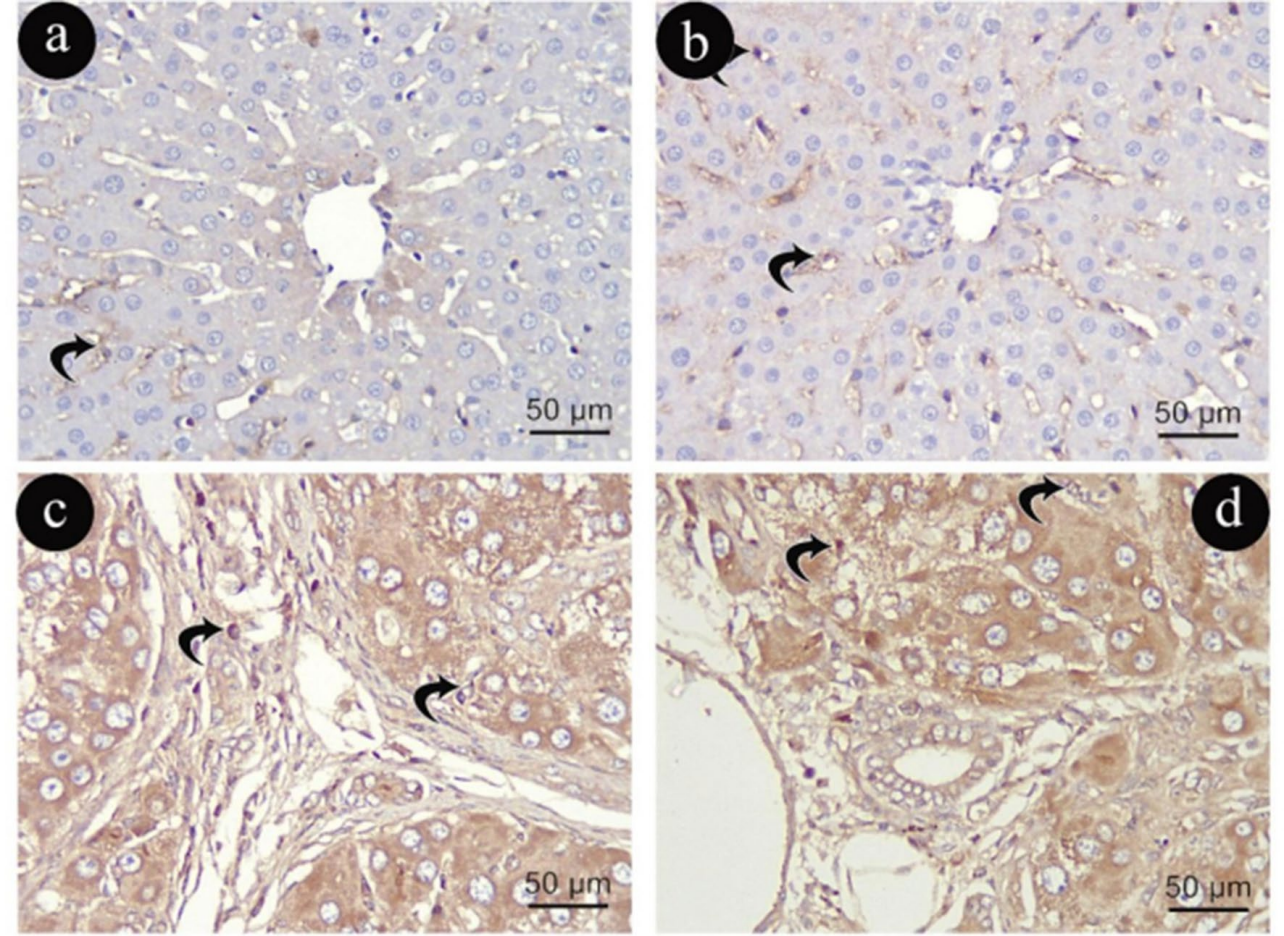
Photomicrographs representing GFAP immune-histochemistry staining of liver sections in group (I)in (**a**&**b**) and (II) in (**c**&**d**). Curved arrows: positive cytoplasmic immune reaction in cytoplasmic processes. (GFAP × 400, scale 50um).

HSCs in Group (I) exhibited a cytoplasmic immunoexpression of GFAP (Fig. 9a, b). As opposed to Group (I), Group (II) had a noticeably higher cytoplasmic positive immunoexpression of GFAP- (Fig. 9 c, d).

**Figure 10:**
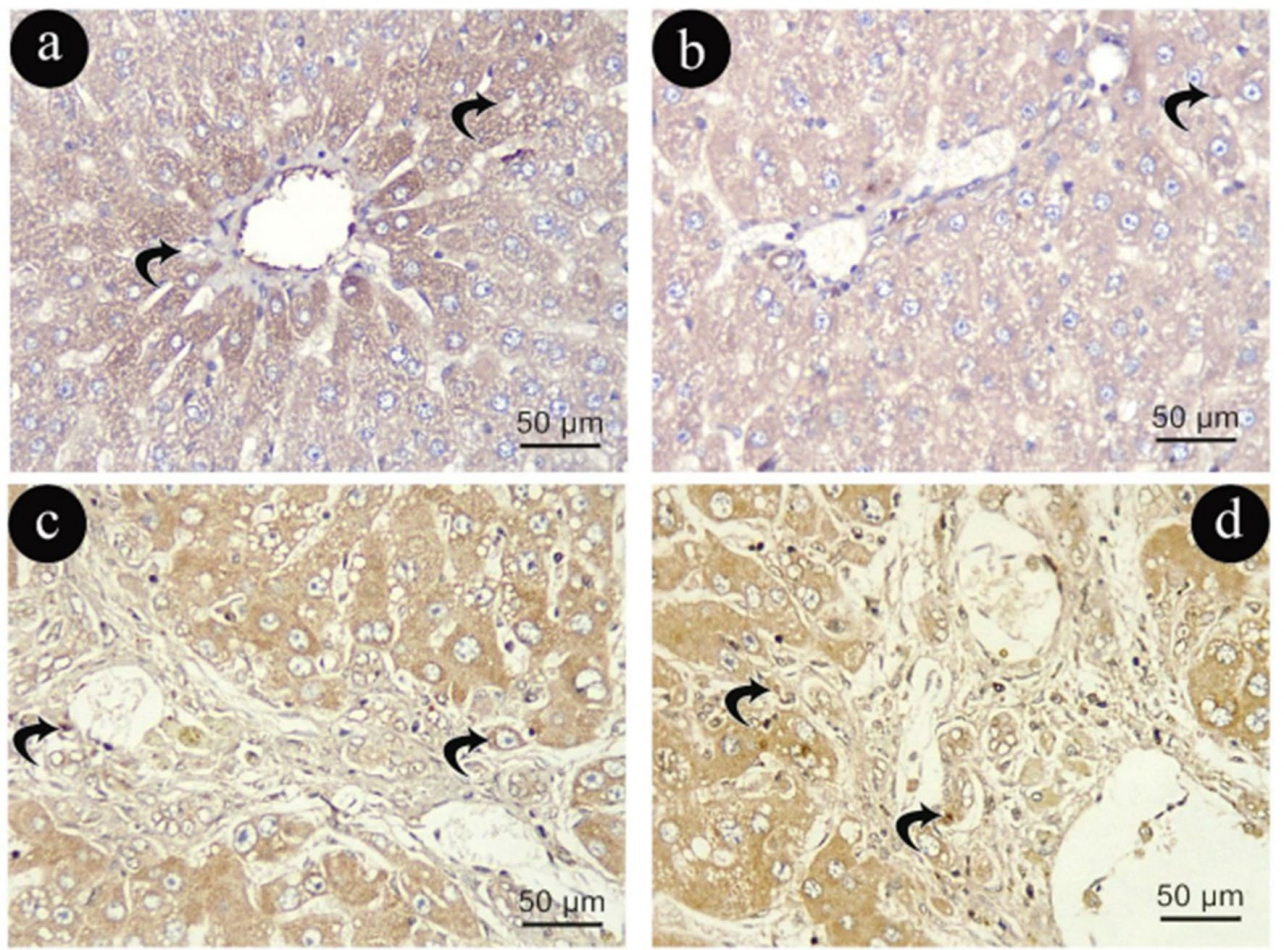
Photomicrographs representing GFAP immune-histochemistry staining of liver sections in group (III) in (**a**&**b**) and (IV) in (**c**&**d**). Curved 222arrows: positive cytoplasmic immune reaction in cytoplasmic processes. (GFAP × 400, scale 50um).

In contrast to Group (I), Group (III) demonstrated widespread of numerous strong GFAP positive cells around portal vein, along sinusoids and in fibrotic lesions with increased number of aged HSCs with mild distribution in GFAP immune-reactive areas (Fig. 10a, b). However, Group (IV) exhibited an apparent increased number and intensity of GFAP positive cytoplasmic immune reaction when compared to Group (III) (Fig. 10c, d).

- TGF-β immune-histochemical staining

**Figure 11:**
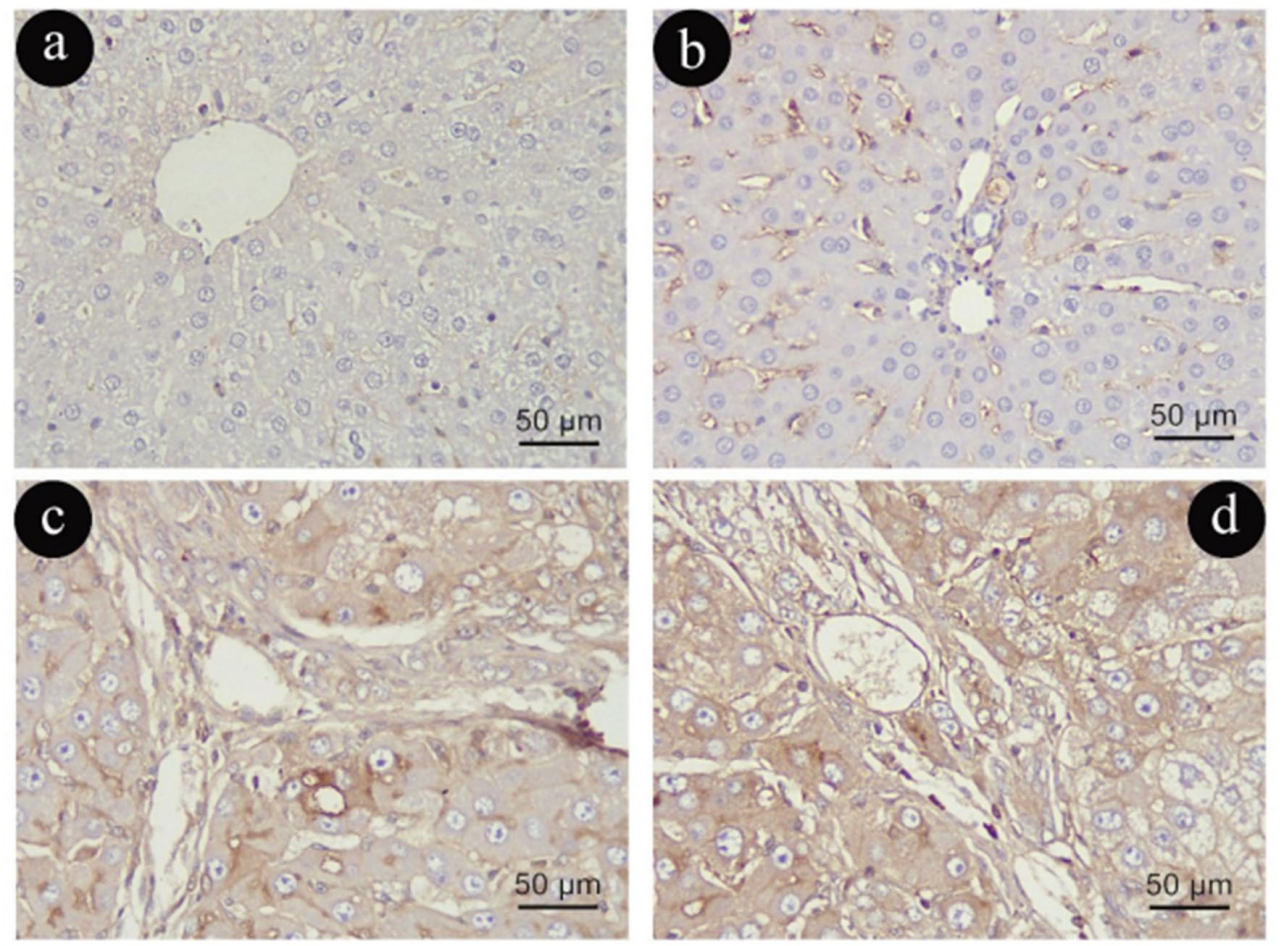
Photomicrographs representing TGF- β immune-histochemistry staining of liver sections in group (I)in (**a**&**b**) and (II) in (**c**&**d**). (TGF-β X400, scale 50um).

TGF-β cytoplasmic immunoreactivity in Group (I) was minimal (Fig. 11a, b). Unlike Group (I), Group (II) showed a significant increase in TGF-B positive immune reaction in hepatic parenchyma. (Fig. 11c, d).

**Figure 12:**
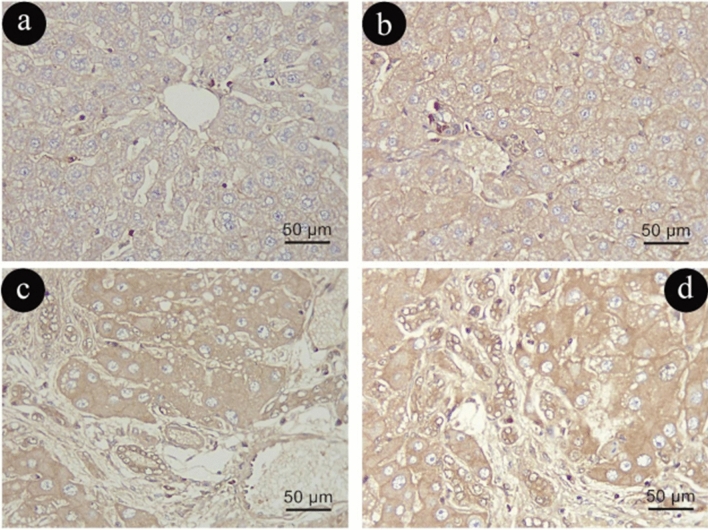
Photomicrographs representing TGF- β immune -histochemistry staining of liver sections in group (III) in (**a**&**b**) and (IV) in (**c**&**d**). (TGF-β X400, scale 50um).

When compared to Group (I), Group (III) showed increased cytoplasmic immunoreactivity against TGF-β due to aging (Fig. 12a, b). The immunoexpression of TGF-β was found to be higher in Group (IV) compared to Group (III) (Fig. 12c, d).

#### Histo-morphometric results

Group (II) demonstrated a statistically significant increase in the mean number of HSC/0.5 mm2, optical density (OD) of α-SMA, area percentage of GFAP, and TGF-β immune-positive expression compared to Group (I). This was evidenced by the results of the histo-morphometric analysis as presented in (Table. 2). Furthermore, there was a statistically significant reduction observed in the four parameters when comparing Group (III) to Group (II). However, the comparison between Group (III) and Group (I) demonstrated a statistically significant increase in the four measures. In addition, a statistically significant increase was observed when comparing Group (IV) to Group (III).

**Table 2:** Histo-morphometric results.

Parameter groups	Group (I)	Group (II)	Group (III)	Group (IV)	*P*-value
Desmin (HSC-count/0.5mm^2^)	19.38 ± 2.973	43.38 ± 2.446^a^	33.00 ± 2.563^b^	53.50 ± 3.586^c^	0.0001**
α-SMA (OD)	0.242 ± 0.0181	0.537 ± 0.063^a^	0.3308 ± 0.006^b^	0.6605 ± 0.054^c^	0.0001**
GFAP (Area %)	5.407 ± 1.553	18.23 ± 1.882^a^	12.31 ± 1.458^b^	33.45 ± 4.012^c^	0.0001**
TGF- β (Area %)	4.954 ± 1.290	21.28 ± 2.062^a^	13.81 ± 1.259^b^	31.51 ± 2.458^c^	0.0001**

All continuous variables were expressed using mean (± SD) and compared using ANOVA test and post hoc LSD test where:

*P* value ≤ 0.0001** was considered statistically highly significant.

^a^ means : significant compared with group I.

^b^ means: significant compared with group I & II.

^c^ means: significant compared with group I, II &III.

n = 8.

### Effect of induction of liver fibrosis on the expression levels of FOXP3, CD133, and TGF- β genes

The expression levels of FOXP3, CD133, and TGF beta genes in rats significantly increased after induction of liver fibrosis, as shown in (Table. 3). We observed a substantial increase in the expression levels of FOXP3, CD133, and TGF- β genes in the liver fibrosis induced groups (II and IV) compared to the normal groups (I and III), respectively (*P* < *0.001)*. FOXP3 and TGF- β expression levels in Group IV reported the highest levels compared to Group II. Nevertheless, the expression level of CD133 was higher in Group II compared to other groups (I, III, and IV) (*P* < *0.001)*. In addition, there was no significant difference between Group III and Group I regarding all examined parameters (Table 4).

**Table 3:** Expression levels of FOXP3, CD133, and TGF- β genes in all studied groups.

	Group 1	Group 2	Group 3	Group 4	F	*P* value
FOXP3	1.08 ± 0.1	5.91 ± 0.48^a^	1.09 ± 0.11^b^	8.37 ± 0.28^a b c^	1184.3	< 0.001 **
CD133	1.04 ± 0.11	7.09 ± 0.15^a^	1.08 ± 0.11^b^	6.56 ± 0.23^a b c^	3449.7	< 0.001 **
TGF- β	1.06 ± 0.09	3.7 ± 0.33^a^	1.11 ± 0.08^b^	5.6 ± 0.34^a b c^	603.1	< 0.001**

All continuous data were expressed using mean (± SD) where:

*P* value ≤ 0.001** was considered statistically highly significant(S).

^a^ means : significant compared with group 1.

^b^ means: significant compared with group 2.

^c^ means : significant compared with group 3.

**Table 4:** Primers used in qRT-PCR.

Gene	Forward	Reverse	pb	Accession no
TGF- β	AGGGCTACCATGCCAACTTC	CCACGTAGTAGACGATGGGC	168	NM_021578.2
GAPDH	GCATCTTCTTGTGCAGTGCC	GGTAACCAGGCGTCCGATAC	91	NM_017008.4
FOXP3	TGATCCCTCTCTGTCAGTCCA	CAGAGTCAGGAAAAGTTGCCG	154	NM_001108250.1
CD133	TGGTCCAGCCGAATGACTTC	AGATGGCGACCTCCTTGGTA	93	NM_021751.2

### Effect of induction of liver fibrosis on hepatic tissue concentration of TGF- β and α-SMA

Regarding TGF-β and α-SMA concentrations in the hepatic tissue as illustrated in (Table 5), there was a significant increase in Group II in comparison to Group I. In addition, there was a non-significant increase in TGF-β concentration in Group III in comparison to Group I while there was significant increase in α-SMA concentration in Group III in comparison to group 1. Moreover, in Group IV there was a significant increase in TGF-β and α-SMA concentrations in comparison to group I, II and III.

**Table. 5:** Hepatic tissue concentration of TGF- β and α-SMA in all studied groups.

	Group I	Group II	Group III	Group IV	F	*P* value
TGF-β1 ng/gm tissue	1.68 ± 0.19	4.24 ± 0.41^a^	2.43 ± 0.36^b^	6.74 ± 1.07^a,b,c^	109.6854	< 0.001**
α-SMA ng/gm tissue	2.21 ± 0.52	10.19 ± 2.13^a^	5.12 ± 1.21^a,b^	16.23 ± 2.56^a,b,c^	94.4671	< 0.001**

All continuous data were expressed using mean (± SD) where:

*P* value ≤ 0.001** was considered statistically highly significant (S).

^a^ means: significant compared with group I.

^b^ means significant compared with group II.

^c^ means: significant compared with group III.

## Discussion

Liver Cirrhosis ranks as the 12th most common cause of death worldwide^27^. One of the primary histological consequences of chronic liver disease is the accumulation of collagen fibers, leading to progressive liver fibrosis and ultimately cirrhosis. The prevalence of liver fibrosis significantly rises with age, with men and women experiencing increased rates from their 40s and 50s, respectively. Among individuals aged 70 or older, the prevalence of significant liver fibrosis reaches 15.3%^28,29^ .

The process of fibrogenesis resulting from liver injury involves intricate interactions among various cell types responding to chronic inflammation, fibrogenesis, and tissue regeneration^25^. Our study, to the best of our knowledge, is the first to elucidate the complex interactome of aging in liver fibrogenesis and regeneration. While the primary function of activated HSC is the synthesis of extracellular matrix proteins, many aspects of this cell type remain unknown^30^.

In our study, we validated liver fibrosis through elevated liver enzymes and the high expression of desmin, α-SMA, GFAP and TGF-β. These findings are consistent with Jeng et al., who reported a positive correlation between α-SMA expression and the degree of fibrosis^31^, while GFAP serves as an early marker of liver fibrosis^32^, and desmin significantly increases in advanced fibrosis^33^. We observed significantly higher expression of both HSC-activation markers, GFAP and desmin, in the fibrosed groups, with group IV exhibiting greater expression compared to group II. On the same side, we made the unexpected observation of elevated expression of fibrosis markers in group III when compared to the control group I. This finding underscores the significant role that aging plays in both the onset and progression of liver fibrosis.

Recent studies have highlighted that stellate cell activation can be beneficial, as it alters gene expression and contributes to liver regeneration through the release of trophic factors^34^. Activated stellate cells are known to promote liver regeneration by releasing retinoids, which support hepatocyte survival and stimulate the release of growth factors, such as Hepatocyte Growth Factor (HGF)^35–37^. HSCs possess stem cell characteristics, including resistance to apoptosis, nestin expression, and CD133 expression, a marker of stem/progenitor cells^30,38^ . While CD133 has been studied as a cancer stem cell marker, its role in stellate cells remains less understood^34^. In our study, we observed a significant increase in CD133 levels, as well as TGF-β, in the fibrosis groups, indicating their potential involvement in liver fibrosis^39^.

Our findings align with Rozeik et al., as we also observed a significant increase in CD133 expression with the inflammatory activity scores and stages of liver fibrosis^40^. However, group II exhibited higher CD133 expression compared to group IV, suggesting a greater regenerative capacity in the former^41^. Notably, Lee et al., emphasized the antifibrogenic role of hepatocyte-CD133 in preventing liver fibrosis by negatively regulating TGF-β^18^.

Kordes et al., proposed that HSCs are progenitor cells with high retinyl palmitate loads^30^ . It was discovered that retinoic acid (RA) controlled the progression of the cell cycle and sped up liver regeneration. This was linked to an early activation of (RA) signaling, as well as elevated expression of receptor binding proteins and retinoid processing enzymes^42^.

The aging process affects the stellate cell niche, potentially explaining its impact on liver fibrosis and regeneration^37^. Aged stellate cells exhibit a senescence-associated secretory phenotype and reduced expression of GFAP. These aging-related changes result in the release of HSCs from their niche and impair the functions of stellate cells by reducing the expression of ECM-associated genes and growth factors like HGF. These factors collectively contribute to the diminished regenerative capacity of aged livers^34^.

The interaction between the immune system and stellate cells is complex, with HSCs displaying immunomodulatory properties similar to mesenchymal stem cells from the bone marrow. In early-stage fibrosis, immune responses contribute to terminating HSC activation, but the interaction of immune mediators in chronic liver disease frequently encourages liver fibrosis^43^.

The activation of Tregs cells during aging is a notable alteration in the adaptive immune response. Tregs has an immunosuppressive function, promoting immune self-tolerance and homeostasis by secreting interleukin-10 (IL-10) and TGF-β. The transcription factor FoxP3 is a distinctive marker of Treg cells and is essential for both the development of Treg and immune control^44^. In our study, we found a selective increase in Treg cells expressing Foxp3 + in the aged fibrotic group compared to the young fibrotic one, and both fibrotic groups are higher than other control groups. This observation is consistent with several previous research^45–47^.

In the present study, we also observed significantly increased levels of Foxp3 and TGFβ in the old fibrotic groups. These findings are in line with the study by Lee et al., which demonstrated that cytokines like IL-10 and TGF-β accumulate with age and induce the transformation of CD4 + CD25-FOXP3- T cells into CD4 + CD25 + FOXP3 + Tregs, which suppress the activation and proliferation of CD4 + and CD8 + T cells^48^.

Aging is associated with changes in the proliferative and functional abilities of the immune system, leading to immunosenescence and inflammation. Th17 cells have been implicated in autoimmune and chronic inflammatory diseases, with a reciprocal relationship between Th17 cells and Tregs^49^ . The Treg/Th17 ratio plays a critical role in intrahepatic immune regulation, and its dysregulation is characteristic of liver fibrosis progression. Schmitt et al. found that the Treg/Th17 ratio decreases with age^49^.

On the other hand, some studies have reported no significant change in Treg cell frequencies during aging, but the capacity of CD4 + FOXP3 + T cells to regulate IL-10 production decreases^50^. There is potential for establishing normal reference ranges of CD4 + CD25 + FOXP3 + Tregs based on different age groups, which could offer new prospects for designing Treg-targeted immunomodulatory strategies^45^.

We observed in our study that elevated cholesterol levels were accompanied by increased liver enzyme (ALT) levels, particularly in the older fibrosed groups compared to their controls. Aligning with this finding, it has been reported that age-related glucose intolerance in humans often coincides with the development of insulin resistance^51^ which serves as one of the underlying mechanisms contributing to elevated levels of very low density lipoprotein (VLDL) cholesterol and low density lipoprotein (LDL) cholesterol. The excessive production of VLDL and triglycerides within the liver has been postulated to stem from elevated serum levels of free fatty acids^52^.

Teratani et al. have previously documented that the accumulation of free cholesterol in HSCs can lead to heightened Toll-like receptors 4 (TLR4) signaling, rendering HSCs more sensitive to TGF-β^53^. Furthermore, cholesterol can activate HSCs through various mechanisms, such as the activation of cholesterol crystal-activated Kupffer cells and oxidized LDL-loaded foam cells, which can induce HSC activation through the release of inflammatory cytokines^54^. Recent research has also demonstrated that oxidized phospholipids can directly trigger the expression of fibrogenic genes in HSCs^55^. Conversely, the study conducted by Kaminsky-Kolesnikov suggests that cholesterol may promote hepatocyte proliferation to mitigate liver scarring by inducing apoptosis in activated HSCs^56^.

Adiponectin, one of the most abundant adipokines, exhibits hepatoprotective and antifibrogenic effects during liver injury. These effects include inhibiting the production of IL-6 and Tumor necrosis factor- α (TNF-α) and HSC proliferation and migration^57^. Our study revealed a significant reduction in adiponectin levels in group IV, characterized by high cholesterol levels and advanced fibrosis, compared to group II. Mohamed et al. and Nazal et al. similarly found a highly significant negative correlation between adiponectin levels and liver fibrosis, with significantly lower adiponectin levels in individuals with high-grade fibrosis^57,58^.

Our study further demonstrated a decrease in adiponectin levels in the older group IV compared to the younger group II, a phenomenon that may be attributed to the presence of insulin resistance, as suggested by Yamauchi et al.^59^.

Several recent reports have highlighted the role of leptin as a critical hormone in the development of liver fibrogenesis, primarily by enhancing the activity of TGF-β1^60^.

The study by Saxena et al. in 2002, investigating the role of leptin in hepatic fibrosis, found that the absence of leptin led to the absence of α-SMA positive cells, underscoring the importance of leptin as a profibrogenic factor in liver fibrosis^61^. Our study also indicated a decreased level of leptin in group IV compared to group II. This decrease may be attributed to aging-related factors, such as the down-regulation of megalin expression, reduced leptin uptake in the hypothalamus due to decreased expression of leptin receptor mRNA, and decreased levels of leptin receptor protein in the hypothalamus, as outlined by Sasaki in 2015^62^.

## Conclusion

Aging had a critical role in the enhancement of liver fibrosis, and there are many underlying mechanisms that may promote this role including and not limited to hormonal changes like adipokines, decreased regenerative capacities and immunity modulation. Hepatic stellate cells were engaged effectively in most of these mechanisms, so we recommend further studies to investigate the crucial intermediate role of hepatic stellate cells as a potential therapeutic target.

## Materials and methods

### Experimental animals

The animals utilized were female Albino rats, weighing 200–230 g each. Standard conditions were prepared for rats to keep them in a temperature (25 ˚C) with 12 h of light and 12 h of darkness and unrestricted access to standard laboratory food and water. This study has been approved by the ethics committee of The Institutional Animal Care and Use Committee, Zagazig University (ZU-IACUC), Egypt (ZU-IACUC /2/F/64/2022). All the procedures and experiments were carried out in accordance with the protocol approved by them, and the animals were handled according to the national and international ethical standards. To keep the experimental animals from experiencing pain or discomfort, this study utilized the earliest endpoint that was scientifically justified. All experiments were performed in accordance with ARRIVE guidelines and regulations.

### Experimental design

Four groups of total 32 albino rats were randomly assigned (8 animals per group): Group I (young “3 months old” with normal liver, n = 8), Group II (young “3 months old” with liver fibrosis, n = 8), Group III (old “18 months old” with normal liver, n = 8) and Group IV (old “18 months old” with liver fibrosis, n = 8). In group II and group IV, induction of fibrosis was done by treating rats two times a week for 8 weeks with thioacetamide (200 mg/kg, ip) (Sigma, St. Louis, MO)^63^ . Blood samples were taken from all groups twenty-four hours after the last dose of medication, and serum was isolated and kept at—20 ˚C for further investigations. Then, rats were anesthetized by intraperitoneal injection of Pentobarbital sodium 50mg/kg and sacrificed for collection of liver samples.

### Serum analysis for detection of cholesterol, adiponectin, leptin and ALT

- Measurement of serum cholesterol level

The serum cholesterol levels were detected by using the commercial kit (SPINREACT, Spain) based on the manufacturer’s instructions. The intensity of the color formed was measured, which is proportional to the cholesterol concentration in the sample.

- Measurement of serum adiponectin and leptin levels

The quantitative determination of rat adiponectin (ADP) and leptin (LEP) concentrations in serum was performed using sandwich ELISA technique as directed by the manufacturer’s instructions (CUSABIO rat adiponectin (ADP) ELISA Kit, USA; Catalog No. CSB E07271r) and (CUSABIO rat leptin (LEP) ELISA Kit, USA; Catalog No. CSBE07433r). The assay ranges were 0.156 ng/ml–10ng/ml and 0.06 ng/ml–50 ng/ml, respectively.

- Measurement of serum ALT

Levels of serum ALT were determined using the commercially available kit (SPINREACT, Spain) in accordance with the manufacturer’s guidelines. Using photometric measurement, the rate of decline in NADH concentration was determined. It is correlated with the amount of ALT present in the sample that is catalytically concentrated.

### Tissue sampling

The rats underwent an overnight fasting by the end of the experiment. The livers were properly dissected, rapidly taken from each group, maintained on ice-cold plates for thirty minutes, and dried after they had been anaesthetized with intraperitoneal injection of Pentobarbital sodium 50mg/kg^64^. All samples from each group were preserved in Bouin's solution and prepared as paraffin blocks for inspection under a light microscope.

### Histological assessment by light microscopic examination

a General histopathological characters by Hematoxylin and eosin stain

The Pathology Department, Faculty of Medicine, Zagazig University, was the site of all procedures. As per routine practices^65^, sections were cut on 5µm thickness, they were dewaxed in xylene, rehydrated in descending grades of alcohol then were stained by H&E.

b Fibrosis Assessment using Masson Trichrome (MT) stain

The Pathology Department, Faculty of Medicine, Zagazig University, was the site of all procedures. As per routine practices^65^, Masson trichrome (MT) stain was used to stain sections that were 5 µm thick that had been placed on glass slides and deparaffinized in xylene.

c *Immunohistochemistry (IHC) for Desmin, α- SMA, GFAP & Transforming Growth Factor –β (TGF-β)*: Desmin, a HSC marker, as well as α-SMA, a marker for activated HSCs, were stained. The liver samples utilized were set in paraffin blocks. Desmin and α-SMA were stained in sections from each block using the Novocastra Novocastra (NCL-DER11 and Novocastra (NCL-SMA) dilutions of 1:200 each). On a Leica Bond Polymer, Refine Detection (DS9800) with Bond DAB enhancer 30 mL (AR9432), rat liver sections were treated according to an automatic IHC Leica staining protocol. For the antigen retrieval stage, which lasted 30 min, EDTA buffer pH 9 was employed to detect desmin. Sections were pretreated with 0.3% hydrogen peroxide for 20 min in PBS and avidin–biotin blocking solutions then overnight primary antibody incubation at 4°C was done. The antimouse biotinylated secondary antibody (1: 150 Sigma, St. Louis, MO) had been applied for 40 min at room temperature then the peroxidase-conjugated streptavidin (1: 50 Sigma, St. Louis, MO) was administered. 3,3-Diaminibenzidine was used to demonstrate peroxidase activity^66^.

For the biotinylated monoclonal mouse antibody for GFAP and the polyclonal anti-mouse antibody for TGF-β, the primary antibody was diluted using PBS at dilutions of 1/500 and 1/100, respectively. The slides were then rinsed with PBS for 5 min before drops of streptavidin peroxidase were applied for 20 min. The slides were chromogenated with diaminobenzidine then treated with distilled water. The slides were then dried, stained with Harris hematoxylin, and cleaned in xylene. Brown color indicated a favorable response. PBS was used as a negative control in place of the main antibody. Universal kits^67^ produced by Biogen Inc. (Cambridge, Massachusetts, USA) for both GFAP and TGF-β were obtained from Dako Company (Egypt).

### Histo-morphometric analysis

A morphometric analysis was performed on each group's rats. At a magnification of 400, 10 non-overlapping fields were captured and examined. Manual counting was done on desmin- positive nucleated cells for each image/0.5mm2. After immunostaining, the density of α-SMA immune-reactive and parenchymal area was determined (large vessels were eliminated) were assessed using optical density (OD), which was acquired by transforming the mean gray level using the following formula: OD = log (256/mean gray level). Following the subtraction of the background, a ratio of the OD of image file was calibrated as % (relative optical density, ROD) using Image J analysis software (Fiji image j; 1.51 n, NIH, USA) at human anatomy and embryology department, Zagazig University.

On the other hand, in GFAP and TGF-β immunohistochemical stained sections, to quantify the percentage area of positive response from 8 rats/group, perceptual fields across the pictures captured by the light microscope at 400 × amplification were selected. To perform a quantitative analysis of the immunoreactivities, stained sections were examined and analyzed by light microscopy (LEICA ICC50 W) in Image Analysis Unit of Human Anatomy and Embryology Department, Faculty of Medicine, Zagazig University.

## Gene expression assessment for FOXP3, TGF-ß and CD133 using real time PCR

### Total RNA extraction from tissue

Total RNA was extracted from homogenized liver tissue using Trizol (Invitrogen; Thermo Fisher Scientific, Inc.) in accordance with manufacturing data. To evaluate the RNA quality, the A260/A280 ratio was analyzed using the NanoDrop® ND-1000 Spectrophotometer (NanoDrop Technologies; Wilmington, Delaware, United States) for 1.5l of the RNA. The estimated purity used for any given RNA was between 1.8 and 2.0. High-Capacity cDNA Reverse Transcription Kit cDNA Kit; (Applied Biosystems™, USA) was used for cDNA synthesis from mRNA extracted in total volume 20 μl as a total of 9 μl containing (1 μg) of RNA sample was added to 11 μL of 2X reverse transcriptase (RT) master mix. then incubated for 60 min at 45 °C, followed by incubation for 10 min at 85 °C to inactivate the enzyme in a Biometra 96-well thermal cycler (Applied Biosystems). The obtained cDNA was stored at 20 °C until it was utilized in subsequent PCR.

### Real – time quantitative PCR (qPCR) analysis

Following the manufacturer's instructions, the real-time RT-PCR was carried out in a Mx3005P Real-Time PCR System (Agilent Stratagene, USA) utilizing TOPrealTM qPCR 2X PreMIX (SYBR Green with low ROX) (Cat. # P725 or P750) (Enzynomics, Korea). In brief, the 20-μL reaction volume included 10 μL TOPreal syberGreen (Enzynomics, Korea), 1 μL each of forward and reverse primer [synthesized by Sangon Biotech, Beijing, China; see (Table. 4) for sequences],1 μL of cDNA, and nuclease-free water up to 20 μL. The PCR cycling conditions included an initial denaturation at 95°C for 12 min followed by 40 cycles of denaturation at 95°C for 20 s, annealing at 60°C for 30 s, and extension at 72°C for 30 s Messenger RNA expression was measured as the fold-change relative to housekeeping genes *Gapdh* compared to the control group following the 2^-ΔΔCT^ method^68^.

### ELISA measurements in tissue homogenate

TFG-β concentration and Tissue a-SMA concentrations in the tissue homogenate were measured using the following rat-specific sandwich enzyme-linked immunosorbent assay kits: Rat TGF-β ELISA Kit (My BioSource, CA, USA) (Cat no: MBS824788) and rat alpha Smooth Muscle Actin ELISA kit (My BioSource, CA, USA) (Cat no: MBS700723). Analyses were performed according to the manufacturers’ instructions.

### Statistical analysis

If the data were normally distributed, continuous variables were expressed as the mean ± SD. The Kolmogorov–Smirnov test was used to pattern normality. To identify significant differences across groups, the one-way ANOVA was used. For several group comparisons, the post-hoc Tukey's test was used. *P* < 0.05 was chosen as the statistical significance cutoff. All statistical calculations were carried out using GraphPad Prism software, version 5.0 (GraphPad Software, San Diego, CA, USA).

### Approval for animal experiments

The study procedures were carried out in accordance with the Declaration of Helsinki. The Institutional Animal Care and Use Committee approved the study (ZU-IACUC /2/F/64/2022).

## Data Availability

The data that support the findings of this study are available from the corresponding author.
